# Dietary Supplementation of Antioxidant Compounds Prevents Light-Induced Retinal Damage in a Rat Model

**DOI:** 10.3390/biomedicines9091177

**Published:** 2021-09-07

**Authors:** Rosario Amato, Alessio Canovai, Alberto Melecchi, Salvatore Pezzino, Roberta Corsaro, Massimo Dal Monte, Dario Rusciano, Paola Bagnoli, Maurizio Cammalleri

**Affiliations:** 1 Department of Biology, University of Pisa, 56127 Pisa, Italy; rosario.amato@biologia.unipi.it (R.A.); a.canovai@student.unisi.it (A.C.); a.melecchi@studenti.unipi.it (A.M.); massimo.dalmonte@unipi.it (M.D.M.); paola.bagnoli@unipi.it (P.B.); 2 Research Center, Sooft Italia SpA, 95100 Catania, Italy; salvatore.pezzino@sooft.it (S.P.); roberta.corsaro@sooft.it (R.C.); dario.rusciano@sooft.it (D.R.); 3 Interdepartmental Research Center Nutrafood “Nutraceuticals and Food for Health”, University of Pisa, 56124 Pisa, Italy

**Keywords:** phototoxicity, oxidative stress, inflammation, neuroprotection, photoreceptor death, electroretinography, nutraceuticals

## Abstract

Light-induced retinal damage (LD) is characterized by the accumulation of reactive oxygen species leading to oxidative stress and photoreceptor cell death. The use of natural antioxidants has emerged as promising approach for the prevention of LD. Among them, lutein and cyanidin-3-glucoside (C3G) have been shown to be particularly effective due to their antioxidant and anti-inflammatory activity. However, less is known about the possible efficacy of combining them in a multicomponent mixture. In a rat model of LD, Western blot analysis, immunohistochemistry and electroretinography were used to demonstrate that lutein and C3G in combination or in a multicomponent mixture can prevent oxidative stress, inflammation, gliotic and apoptotic responses thus protecting photoreceptor cells from death with higher efficacy than each component alone. Combined efficacy on dysfunctional electroretinogram was also demonstrated by ameliorated rod and cone photoreceptor responses. These findings suggest the rationale to formulate multicomponent blends which may optimize the partnering compounds bioactivity and bioavailability.

## 1. Introduction

Oxidative stress and exacerbated inflammatory response are key pathological mechanisms driving several high-incidence neurodegenerative disorders of the retina [1]. In particular, under excessive light irradiation and/or dysfunction of the visual cycle, photoreceptors accumulate toxic metabolites promoting the overproduction of reactive oxygen species (ROS) [2,3]. ROS overproduction triggers an endogenous antioxidant response mediated by the increase in nuclear factor erythroid 2-related factor 2 (Nrf2) with the consequent increment in the production of antioxidant enzymes such as heme oxygenase-1 (HO-1) [4]. In some cases, the accumulation of oxidative species overcomes the protective potential of antioxidant responses thus producing an oxidative imbalance and the onset of oxidative stress [5].

Several promising treatments preventively targeting oxidative stress-driven neurodegeneration have emerged in recent years [6]. Among them, the administration of natural substances by dietary supplementation has been increasingly considered for their potential antioxidant and anti-inflammatory efficacy attained with a low invasiveness [7]. Several studies have suggested that treatment with protective compounds that inhibit oxidative stress and inflammation could reduce the amount of photoreceptor cell death in animal models of light-induced retinal damage (LD) [8,9,10]. In particular, exogenous compounds belonging to carotenoid or anthocyanin families, such as lutein and cyanidin-3-glucoside (C3G), have been recognized among the most promising substances capable to prevent/counteract oxidative stress through a direct free radical scavenging effect [11,12].

Lutein is a dietary carotenoid found in several fruits and vegetables that accumulates in the human retina at the macular level [13,14]. Besides having anti-oxidative and anti-inflammatory properties [15,16], lutein is also able to filter blue light thus protect photoreceptors from light-induced damage [17]. C3G is one of the most common anthocyanins naturally found in many edible parts of plants and appears to benefit vision through its potent antioxidant and anti-inflammatory activity [18,19]. Additional efficacy of C3G depends on its capability to stimulate rhodopsin regeneration in the outer retina [20]. In response to oxidative stress, the antioxidant and anti-inflammatory properties of lutein or C3G result in improved retinal function presumably through reduced photoreceptor death [11,12].

Considering the protective potential of lutein or C3G in models of photooxidative damage, their possible administration in a coupled formulation or in the context of a multicomponent antioxidant mixture would represent an interesting treatment proposal. In this respect, the combination of different antioxidants has been proven as a promising approach for the treatment of retinal neurodegenerative diseases [6]. The use of mixed natural compounds may exert a synergistic effect because of their ability to affect multiple targets [21]. This has been particularly evident in the treatment of photoreceptor degeneration in the rd1 model of retinitis pigmentosa in which the use of individual antioxidants had no significant rescue effect, while treatment with a combination drastically reduced rod degeneration [22].

In the present study, we address the antioxidant efficacy of lutein and C3G administered either in combination or in the context of a pre-formulated multicomponent mixture in a rat model of LD (LD rats) that mimics photoreceptor loss induced by oxidative stress. This model is characterized by an increased expression of proinflammatory chemokines, which further enhance oxidative stress and activate microglia to clear dying photoreceptors [23,24]. In LD rats, we evaluated the preventive efficacy of lutein and C3G administered either alone, in combination or in a multicomponent mixture on oxidative stress, inflammation, gliotic responses, apoptotic markers and photoreceptor cell death. In addition, preventive efficacy on dysfunctional electroretinogram (ERG) was also investigated with a particular focus on the relative contributions of rod and cone photoreceptors.

## 2. Materials and Methods

### 2.1. Animals

Animals were used in compliance with the Association for Research in Vision and Ophthalmology statement for the Use of Animals in Ophthalmic and Vision Research. The present study also adheres to the European Communities Council Directive (2010/63/UE) and the Italian guidelines for animal care (DL 26/14). The experimental protocol was approved by the Commission for Animal Wellbeing of the University of Pisa (protocol no. 133/2019-PR, 14 February 2019). According to the 3Rs principles for ethical use of animals in scientific research, all efforts were made to reduce both the number of animals and their suffering. Male Sprague Dawley rats (8 weeks old) were obtained from Envigo Italy (San Pietro al Natisone, Italy). Animals were maintained in a regulated environment (23 ± 1 °C, 50 ± 5% humidity) with 12 h light/dark cycles (lights on at 08:00 a.m.) and fed with a standard diet and water ad libitum. 50 rats were used. Of them, eight were used for lutein and C3G detection in the plasma and ocular tissues (see below) and six were used as control group. The remaining 36 rats underwent to LD protocol (see below) and were divided in six experimental groups (six rats in each experimental group): LD untreated group, lutein group (+Lut), C3G group (+C3G), lutein/C3G group (+Lut/C3G), low dose mixture group (+low dose) and high dose mixture group (+high dose).

### 2.2. Treatments

Lutein oil from *Tagetes erecta* titrated at 20% lutein was obtained from Procemsa (Torino, Italy). Black rice extract titrated at 20% in cyanidine-3-glucoside and verbascoside from *Verbascum Thapsus* titrated at 10% were obtained from “La sorgente del Benessere” (Fiuggi, Italy). Treatments were performed by oral gavage once daily for 7 days before undergoing the LD protocol. Once a day, the compounds, either individually or combined were freshly prepared by dissolving each compound in wheat germ oil to obtain the desired final dose 5.2 mg/Kg lutein and 4.0 mg/Kg C3G administered individually or in combination. In a second set of experiments, based on different commercial formulations for humans, a nutraceutical blend containing lutein, C3G, verbascoside and zinc (hereinafter referred to as mixture) was obtained by Sooft, SpA, Montegiorgio, Italy. The mixture was administered at two dosages in mg/Kg: 1.08 lutein, 3.6 C3G; 0.36 verbascoside and 0.88 zinc (low dose) or 3.24 lutein, 10.8 C3G, 1.08 verbascoside and 2.64 zinc (high dose). These doses correspond to the recommended in humans normalized by the body surface area method for interspecies’ drug dosage translation [25].

### 2.3. Determination of Lutein and C3G

After 7 days of lutein or C3G administration, animals were euthanized, plasma samples were collected and choroid and retina were isolated from their eyes. Pooled tissue samples of either choroid or retina were homogenized with an Ultra-Turrax homogenizer for 3 min on ice at 4 °C in methanol as extraction solvent (1 µL per mg of tissue), then subjected to a 5-min ultrasonication in a cold-water bath. A volume of 100 µL of plasma was extracted with 100 µL of methanol and sonicated. All samples were finally centrifuged for 10 min at 10,000 rpm and the supernatants were stored frozen for further analysis. Lutein and cyanidin 3-glucoside were quantitated in tissue extracts by HPLC/MS/MS using the triple quadrupole instrument Agilent 6410-A equipped with a Phenomenex Gemini C18 column at 25 °C under isocratic conditions using 10% of Buffer A (water 0.5% formic acid) and 90% of methanol at a flow rate of 0.1 mL/min. The system is equipped with a positive ionizing mode ESI interface, such that the mass transition for lutein is 551 > 429 *m*/*z* and for C3G is 449 > 287 *m*/*z*. The operational MS parameters of the instrument were: gas temperature, 350 °C; gas flow, 6 L/min; nebulizer, 20 psi; capillary, 4000 V; collision energy, 15 V; dwell-time, 200 msec; fragmentor, 135 V. Calibration curves were constructed adding each pure compound to the respective blank tissue extract, devoid of detectable amounts of lutein and C3G.

### 2.4. Light-Induced Damage

The protocol used here for light-induced damage is in line with that reported in previous studies in which the duration and the intensity of light exposure needed to cause morphological and functional damage to the retina have been determined [26]. In particular, rats were dark-adapted overnight and then their pupils were dilated by 1% tropicamide eye drops (Allergan S.p.A., Rome, Italy) immediately before the light exposure. Each animal was kept separately in a small cage and placed in the middle of a custom-made light box apparatus (dimensions 72 × 61 × 52 cm). Light intensity was measured through a digital illuminance meter (Dr. Meter, Ahern Ave, Union City, CA, USA). Rats were exposed to diffused 1000 lux cool-white light continuatively for 24 h emitted from six light-emitting diode bulbs. The temperature of the apparatus was controlled by a cooling aeration system based on a small air fan and maintained at (25 ± 1.5 °C). After light exposure, the rats were returned to the dim cyclic light environment. Two days later, rats underwent to electroretinography (ERG) and subsequent sacrifice by a lethal dose of sodium pentobarbital.

### 2.5. Electroretinography

Control and LD rats were dark-adapted overnight and anesthetized by intraperitoneal injection of 30 mg/kg sodium pentobarbital. Pupils were dilated with a topical drop of 1% tropicamide (Allergan S.p.A.) and a heating pad was used to keep the body temperature at 37.5 °C. The electrophysiological signals were recorded through silver/silver chloride corneal ring electrodes inserted under the lower eyelids avoiding visual field obstruction. Saline solution drops were intermittently instilled to prevent ocular surface dryness and clouding. Each corneal electrode was referred to a needle electrode inserted subcutaneously at the level of the corresponding frontal region. The ground electrode was inserted subcutaneously at the tail root. The scotopic responses were evoked by a 10 cd-s/m^2^ flash intensity delivered with a Ganzfeld stimulator (Biomedica Mangoni, Pisa, Italy). After the scotopic stimulation, rats were adapted to a background light intensity of 30 cd/m^2^ for 10 min. Photopic, cone-mediated responses, were recorded at 3 cd-s/m^2^ flash stimuli. The average of 20 consecutive responses was considered for the photopic ERG analyses. The interval between light flashes was adjusted to appropriate times that allowed response recovering (20 s for scotopic responses, 3 s for photopic responses). Responses were collected simultaneously from both eyes, amplified at 1000 gain, and filtered with a bandpass of 0.2 to 500 Hz before being digitized at 5 kHz rate with a data acquisition device (Biomedica Mangoni). All ERG waveforms were analyzed using a customized program (Biomedica Mangoni). Noise amplitude was evaluated by measuring the electrical activity in absence of light stimuli. In compliance with the International Society for Clinical Electrophysiology guidelines, the b-wave amplitude was measured from the trough of the a-wave to the peak of the b-wave or, if no a-wave was detectable, from the pre-stimulus baseline.

### 2.6. Immunohistochemistry

Eyeballs were immersion-fixed in 4% paraformaldehyde in 0.1 M phosphate-buffered saline (PBS) for 2 h at room temperature, transferred to 25% sucrose in 0.1 M PBS and stored at 4 °C. After being embedded in cryo-gel medium, fixed eyes were cut into 10 µm thick coronal sections and mounted onto positive charged slides. Immunostaining was performed by incubating mounted sections with mouse monoclonal anti-rhodopsin (ab5417, Abcam, Cambridge, UK; dilution 1:200), rabbit polyclonal anti-cone arrestin (AB15282, Sigma-Aldrich, St. Louis, MO, USA; dilution: 1:200), rabbit monoclonal anti-glial fibrillary acid protein (GFAP; ab207165, Abcam; dilution: 1:400) and rabbit monoclonal anti-ionized calcium-binding adapter molecule 1 (Iba-1; ab178846, Abcam; dilution: 1:200) antibodies diluted in 0.1% *v/v* Triton X-100 in 0.1 M PBS overnight at 4 °C. After being rinsed, mounted sections were incubated with appropriate goat polyclonal anti-mouse conjugated with Alexa-Fluor 488 (A-11001, Thermo Fisher Scientific, Waltham, MA, USA; dilution: 1:200), goat polyclonal anti-rabbit conjugated with Alexa-Fluor 555 (ab150078, Abcam; dilution: 1:200) or goat polyclonal anti-rabbit conjugated with Alexa-Fluor 488 (ab150077, Abcam; dilution: 1:200) secondary antibodies diluted in 0.1% *v/v* Triton X-100 in 0.1 M PBS for 2 h at room temperature. Then, retinal sections were coverslipped with Fluoroshield mounting medium containing 4′, 6-diamidino-2-phenylindole (DAPI; Abcam). The image acquisition was performed using an epifluorescence microscope (Ni-E; Nikon-Europe, Amsterdam, The Netherlands) equipped with a 20× plan apochromat objective and a digital camera (DS-Fi1 c; Nikon-Europe). Levels of the immunohistochemical signal were quantified by averaging the fluorescence intensity of five coronal sections randomly chosen from each retina (six retinas per group). Four images per section were quantified by using the analysis tool of Adobe Photoshop. Quantification was performed in a masked manner. Images were then turned into grayscale, normalized for the background and analyzed for the mean gray levels to quantify the immunofluorescence intensity for each marker. The outer nuclear layer (ONL) thickness (extending from the interface between the outer plexiform layer and the inner segment layer) was measured in five sections for each retina (six retinas per group) and four images in each section were sampled.

### 2.7. Western Blot

Eyes were enucleated, retinas were dissected and stored at −80 °C. Samples were lysed with RIPA lysis buffer (Santa Cruz Biotechnology, Dallas, TX, USA) supplemented with proteinase and phosphatase inhibitor cocktails (Roche Applied Science, Indianapolis, IN, USA). Protein content was quantified by Micro BCA Protein Assay (Thermo Fisher Scientific, Waltham, MA, USA). Samples containing 30 µg of proteins were subjected to SDS-PAGE (4–20%; Bio-Rad Laboratories, Inc, Hercules, CA, USA) and gels were transblotted onto nitrocellulose membranes (Bio-Rad Laboratories, Inc). After a blocking phase with either 5% skim-milk or 4% bovine serum albumin (BSA), membranes were incubated overnight at 4 °C with the primary antibodies listed in Table 1. Then, blots were incubated for 2 h at room temperature with appropriate HRP-conjugated secondary antibodies (rabbit anti-goat, sc-2768, Santa Cruz Biotechnology; rabbit anti-mouse, A9044, Sigma-Aldrich; goat anti-rabbit, 170–6515, Bio-Rad Laboratories, Inc; all at 1:5000). Blots were developed by the Clarity Western enhanced chemiluminescence substrate (Bio-Rad Laboratories, Inc). Blot images were acquired with the ChemiDoc XRS+ (Bio-Rad Laboratories, Inc). The optical density (OD) of the target bands (Image Lab 3.0 software; Bio-Rad Laboratories, Inc) were normalized for the relative OD of β-actin as a loading control or nuclear factor kappa-light-chain-enhancer of activated B cells (NF-κB) p65 as appropriate. Although their reactivity with rat antigens is not reported, cleaved caspase 3, Nfr2 and NF-kB antibodies are apparently selective also for rats as reported in the operating instructions of the customer. This is also in line with previous studies in which these antibodies were used in rat samples [27,28].

### 2.8. Statistical Analysis

Statistical analyses were performed using the Graph Pad Prism 8.0.2 software (GraphPad Software, Inc, San Diego, CA, USA). Differences between groups were tested using two-tailed *t*-test or one-way ANOVA followed by Newman–Keuls multiple comparison post hoc test. Differences with *p* < 0.05 were considered significant. All data are expressed as mean ± SD of the indicated *n* values.

## 3. Results

### 3.1. Plasma, Choroid and Retinal Levels of Lutein and C3G

**Figure 1:**
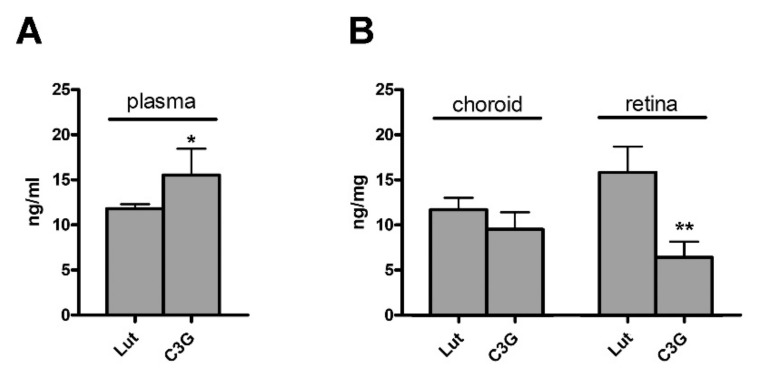
Plasma, choroid and retina concentration of lutein and C3G after 1 week of administration. (**A**) Mean total lutein and C3G concentration measured in plasma. (**B**) Mean total lutein and C3G concentration measured in choroid and retina. Data are expressed as mean ± SD (*n* = 4). Differences between groups were tested for statistical significance using two-tailed t-test. * *p* < 0.05; ** *p* < 0.01 versus lutein.

After 7 days of administration of either lutein (5.2 mg/Kg) or C3G (4.0 mg/Kg), their levels were measured in plasma, choroid and retina in order to establish whether both compounds reached the choroidal vascular plexus to be conveyed to the retina. As shown in Figure 1A, plasma levels of lutein (11.8 ± 0.5 ng/mL) were lower than those of C3G (15.56 ± 2.9 ng/mL; *p* = 0.044). Lutein concentration was higher in the retina than in the choroid (15.9 ± 3.1 vs. 11.7 ± 1.1 ng/mg), while the opposite occurred for C3G (6.4 ± 1.4 vs. 9.47 ± 2.1 ng/mg). Choroid levels of lutein and C3G were similar, while retinal levels of lutein were higher than those of C3G (*p* = 0.0017; Figure 1B).

### 3.2. Combined Efficacy of Lutein and C3G on Oxidative Stress

**Figure 2:**
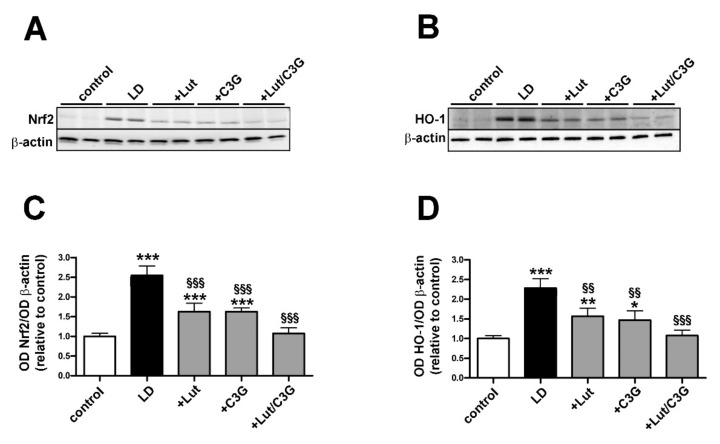
Individual and combined effects of lutein and C3G on oxidative stress markers. (**A**,**B**) Representative Western blots from retinal homogenates of control and LD rats untreated or pretreated with either lutein, C3G or their combination. (**C**,**D**) Densitometric analysis of nuclear factor erythroid 2-related factor 2 (Nrf2) and heme oxygenase-1 (HO-1) immunoblots. The expression of Nrf2 and HO-1 was normalized to the loading control β-actin. Data are expressed as mean ± SD (*n* = 6). Differences between groups were tested for statistical significance using one-way ANOVA followed by the Newman–Keuls multiple comparison post-hoc test. * *p* < 0.05; ** *p* < 0.01; *** *p* < 0.001 versus control; ^§§^ *p* < 0.01; ^§§§^ *p* < 0.001 versus LD.

As shown in Figure 2, in untreated LD rats, protein levels of Nrf2 were upregulated in response to light stimulation (control 1.0 ± 0.075; LD 2.4 ± 0.24; *p* < 0.0001). As a consequence of Nrf2 upregulation, HO-1 was increased, as compared to control rats (control 1.0 ± 0.07, LD 2.12 ± 0.24; *p* < 0.0001). Compared to untreated LD rats, pretreatment with either lutein or C3G resulted in a 40% decrease in the protein levels of Nrf2 (+Lut 1.63 ± 0.16, *p* < 0.0001; +C3G 1.62 ± 0.098, *p* < 0.0001) and HO-1 (+Lut 1.56 ± 0.2, *p* = 0.0070; +C3G 1.46 ± 0.24, *p* = 0.0017), which still remained about 50 % higher than in controls (+Lut *p* = 0.0059; +C3G *p* = 0.025). The pretreatment with lutein in combination with C3G resulted in a much stronger decrease in Nrf2 (+Lut/C3G 1.06 ± 0.14, *p* < 0.0001) and HO-1 levels (+Lut/C3G 1.07 ± 0.13, *p* < 0.0001) which returned to levels similar to those measured in control rats.

### 3.3. Combined Efficacy of Lutein and C3G on Inflammatory Response

**Figure 3:**
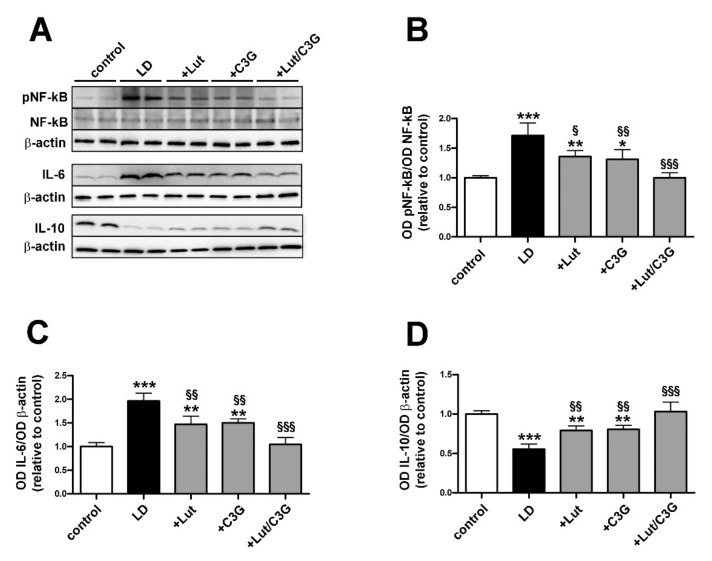
Individual and combined effects of lutein and C3G on inflammatory markers. (**A**) Representative Western blots. (**B**–**D**) Densitometric analysis of the phosphorylation levels of nuclear factor kappa-light-chain-enhancer of activated B cells (NF-kB), interleukin (IL)-6 and IL-10. The expression of pNF-kB was normalized to the level of NF-kB, while the expression of IL-6 and IL-10 was relative to the loading control β-actin. Data are expressed as mean ± SD (*n* = 6). Differences between groups were tested for statistical significance using one-way ANOVA followed by the Newman–Keuls multiple comparison post hoc test. * *p* < 0.05; ** *p* < 0.01; *** *p* < 0.001 versus control; ^§^ *p* < 0.05; ^§§^ *p* < 0.01; ^§§§^ *p* < 0.001 versus LD.

As shown in Figure 3, LD rats displayed upregulated levels of pro-inflammatory markers including the phosphorylated form of the nuclear factor kappa-light-chain-enhancer of activated B cells (NF-kB; LD 1.76 ± 0.21, *p* < 0.0001) and interleukin-6 (IL-6; LD 1.96 ± 0.16, *p* < 0.0001), while the anti-inflammatory cytokine IL-10 was downregulated (LD 0.55 ± 0.06, *p* < 0.0001). Pretreatment with either lutein or C3G reduced by about 25% both phosphorylated NF-kB (+Lut 1.39 ± 0.09, *p* = 0.0106; +C3G 1.33 ± 0.15, *p* = 0.0029) and IL-6 (+Lut 1.47 ± 0.17, *p* = 0.0014, +C3G 1.50 ± 0.08, *p* = 0.0024) in concomitance with a 60% increase in IL-10 levels (+Lut 0.79 ± 0.06, *p* = 0.0034; +C3G 0.80 ± 0.05, *p* = 0.0020) as compared to untreated LD rats. After pretreatment with lutein and C3G, the levels of pro-inflammatory NF-kB (+Lut/C3G 0.99 ± 0.08, *p* = 0.98) and IL-6 (+Lut/C3G 1.04 ± 0.14, *p* = 0.97) and anti-inflammatory IL-10 (+Lut/C3G 1.03 ± 0.11, *p* = 0.98) did not differ from those measured in controls.

### 3.4. Combined Efficacy of Lutein and C3G on Gliosis and Microglial Activation

**Figure 4:**
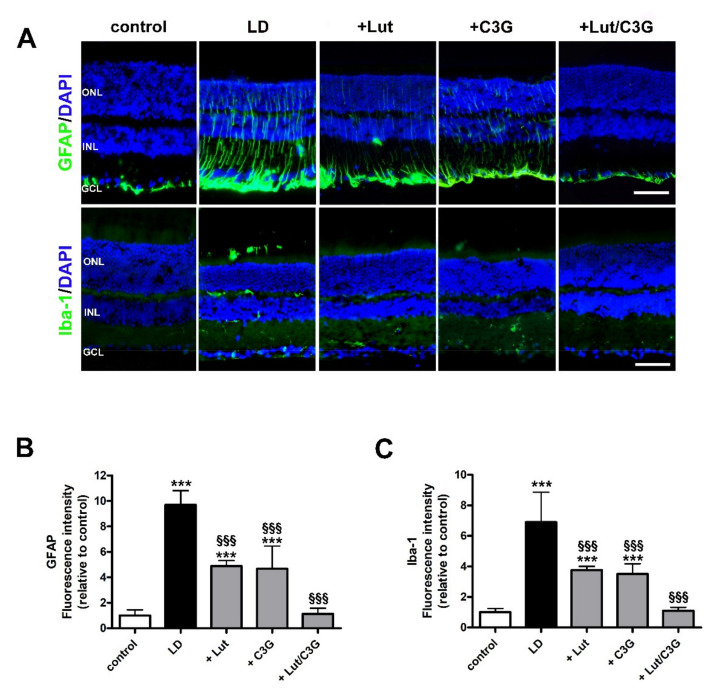
Individual and combined effects of lutein and C3G on gliosis and microglial activation. (**A**) Representative images of retinal sections from control and LD rats untreated or pretreated with either lutein, C3G or their combination immunolabeled for glial fibrillary acidic protein (GFAP; green) and ionized calcium binding adaptor molecule 1 (Iba-1; green). Sections are counterstained with DAPI to highlight retinal nuclear layers. (**B**,**C**) Quantitative analysis of fluorescence intensity. GCL, ganglion cell layer; INL, inner nuclear layer; ONL, outer nuclear layer. Scale bar, 50 µm (*n* = 6 retinas per group). Data are expressed as mean ± SD. Differences between groups were tested for statistical significance using one-way ANOVA followed by the Newman–Keuls multiple comparison post hoc test. *** *p* < 0.001 versus control; ^§§§^ *p* < 0.001 versus LD.

Oxidative stress and inflammation promote Müller cell gliosis as characterized by GFAP upregulation. In addition, the activation of microglia, as identified by Iba-1 upregulation, further contributes to the inflammatory response. As shown in Figure 4A, in control retinas, GFAP immunoreactivity was confined to the ganglion cell layer (GCL) while in untreated LD rats, Müller cells showed extensive GFAP immunolabeling along their processes spreading across retinal layers. GFAP-positive processes were less evident, but still detectable in retinas of lutein- or C3G-treated rats. On the contrary, in retinas of rats pretreated with lutein and C3G in combination, GFAP immunoreactivity in vertical processes was undetectable resulting confined to the GCL, similarly to controls. Quantitative analysis of fluorescence intensity showed that in untreated LD rats, GFAP immunoreactivity was increased as compared to controls (LD; 9.70 ± 1.12, *p* < 0.0001). Individual administration of lutein or C3G reduced GFAP intensity (+Lut; 4.88 ± 0.45, *p* < 0.0001; +C3G; 4.68 ± 1.79, *p* < 0.0001) with no difference among single compounds. In contrast, the pretreatment with their combination resulted in a further decrease of GFAP immunofluorescence that became 85% lower than in untreated LD rats (+Lut/C3G; 1.13 ± 0.44, *p* < 0.0001) with no statistical difference from control rats (*p* = 0.98; Figure 4B). As shown in Figure 4A, Iba-1 immunolabeling was weakly represented in the inner retinal layers of control retinas, with arborizing processes likely associated to inactive microglia. In contrast, Iba1 immunoreactivity was more prominent in the retina of untreated LD rats with enlarged amoeboid-shaped immunopositive cells localized in INL, ONL and subretinal space. Pretreatment with either lutein or C3G partially prevented microglial activation as determined by decreased Iba1-immunolabeled cells, while no evidence of activated microglia was observed when the compounds were given in combination. As shown by the quantitative analysis of fluorescence intensity, in untreated LD rats, Iba-1 was increased compared to controls (LD; 6.90 ± 1.96, *p* < 0.0001). Iba1 immunofluorescence was reduced by about 47% after the administration of either lutein or C3G compared to LD rats (+Lut 3.76 ± 0.24, *p* = 0.0001; +C3G 3.50 ± 0.67, *p* < 0.0001), while their combination preserved Iba1 immunoreactivity to control levels (*p* = 0.95; Figure 4C).

### 3.5. Combined Efficacy of Lutein and C3G on Photoreceptor Degeneration

**Figure 5:**
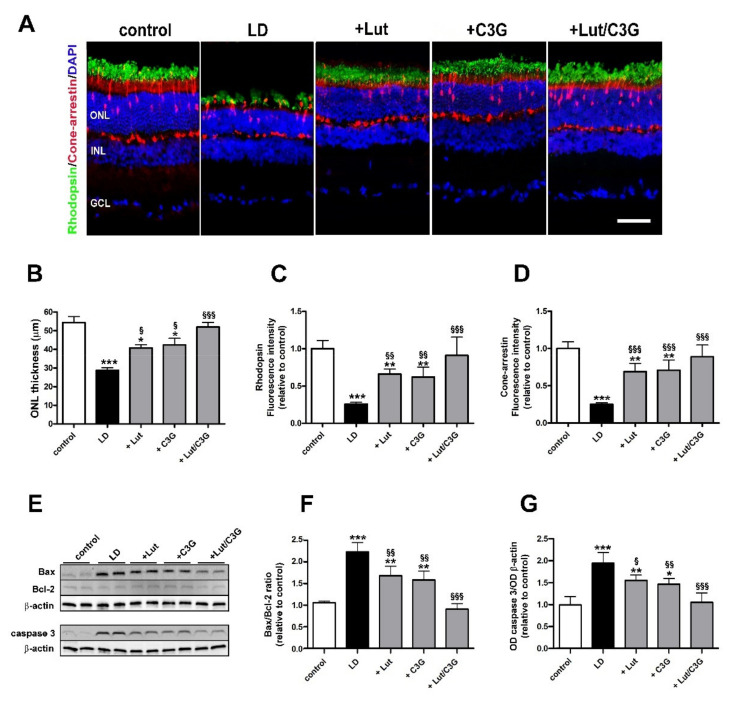
Individual and combined effects of lutein and C3G on photoreceptor degeneration. (**A**) Representative images of retinal sections from control and LD rats untreated or pretreated with either lutein, C3G or their combination immunolabeled for rhodopsin (green) and cone-arrestin (red). Retinal nuclear layers are highlighted by the DAPI counterstaining. (**B**) Quantitative analysis of ONL thickness. (**C**,**D**) Quantitative analysis of rhodopsin and cone-arrestin immunofluorescence intensity. GCL, ganglion cell layer; INL, inner nuclear layer; ONL, outer nuclear layer. Scale bar, 50 µm (*n* = 6 retinas per group). (**E**) Representative Western blots of Bax, Bcl-2 and active caspase 3 from each experimental group. (**F**,**G**) Densitometric analysis of the Bax/Bcl-2 ratio and active caspase 3. The expression of active caspase 3 was relative to the loading control β-actin. Data are expressed as mean ± SD. Differences between groups were tested for statistical significance using one-way ANOVA followed by the Newman-Keuls multiple comparison post-hoc test. * *p* < 0.05; ** *p* < 0.01; *** *p* < 0.001 versus control; ^§^ *p* < 0.05; ^§§^ *p* < 0.01; ^§§§^ *p* < 0.001 versus LD.

Representive images of retinal sections immunolabeled with rhodopsin or cone arrestin and counterstained with DAPI are shown in Figure 5A. Retinas of untreated LD rats showed a prominent decrease ONL thickness compared to control (LD 28.8 ± 3.03 µm, *p* < 0.0001; Figure 5B), as a consequence of photoreceptor loss. Photoreceptors were partially protected following individual pretreatment with lutein or C3G compared to LD (+Lut 40.80 ± 4.02 µm, *p* = 0.027; +C3G 42.40 ± 7.3 µm, *p* = 0.010). No evidence of photoreceptor loss was observed after combined pretreatment (+Lut/C3G 52.00 ± 5.38 µm, *p* = 0.96). Untreated LD rats showed a similar residual expression of rhodopsin (LD 0.26 ± 0.02, *p* < 0.0001) and cone arrestin (LD 0.25 ± 0.02 *p* < 0.0001) compared to control. After individual treatments with lutein or C3G, the immunofluorescence levels of both rhodopsin (+Lut 0.66 ± 0.06, *p* = 0.0016; +C3G 0.62 ± 0.13, *p* = 0.0044) and cone arrestin (+Lut 0.69 ± 0.11, *p* < 0.0001; +C3G 0.71 ± 0.13, *p* < 0.0001) were partially preserved compared to those in LD rats. In contrast, retinas of light-exposed rats treated with lutein and C3G in combination displayed a well-organized and abundant expression of rhodopsin (+Lut/C3G 0.91 ± 0.24, *p* = 0.84) and cone arrestin (+Lut/C3G 0.89 ± 0.15, *p* = 0.55) at levels similar to control (Figure 5C,D).

We investigated whether the dietary combination of lutein and C3G might affect the activation of selected components in the apoptotic pathway that is known to be dysregulated in LD rats [29]. A major checkpoint in the apoptotic pathway is the ratio of pro-apoptotic (Bax) to anti-apoptotic (Bcl-2), playing a key role in the apoptotic pathway. As shown by the representative blots in Figure 5E and the densitometric analysis in Figure 5F,G the Bax/Bcl-2 ratio was increased in untreated LD rats compared to control (LD 2.23 ± 0.21, *p* < 0.0001) with upregulated levels of the pro-apoptotic Bax and stable levels of the anti-apoptotic Bcl-2 (Figure 5F). Downstream to the increased Bax/Bcl-2 ratio, levels of caspase 3 increased in LD rats compared to control (LD 1.96 ± 0.24, *p* < 0.0001; Figure 5G). Treatment with lutein or C3G attenuated the increase in Bax/Bcl-2 ratio (+Lut 1.68 ± 0.22, *p* = 0.0048; +C3G 0.58 ± 0.20, *p* = 0.0010) and the caspase level (+Lut 1.56 ± 0.12, *p* = 0.048; +C3G 1.47 ± 0.13, *p* = 0.018) compared to LD untreated rats. The combined treatment further reduced the levels of Bax/Bcl-2 ratio (+Lut/C3G 0.90 ± 0.12, *p* < 0.0001) and caspase 3 (+Lut/C3G 1.06 ± 0.21, *p* < 0.0001) compared to LD rats, reaching values that were comparable to those measured in controls.

### 3.6. Combined Efficacy of Lutein and C3G on Retinal Function

**Figure 6:**
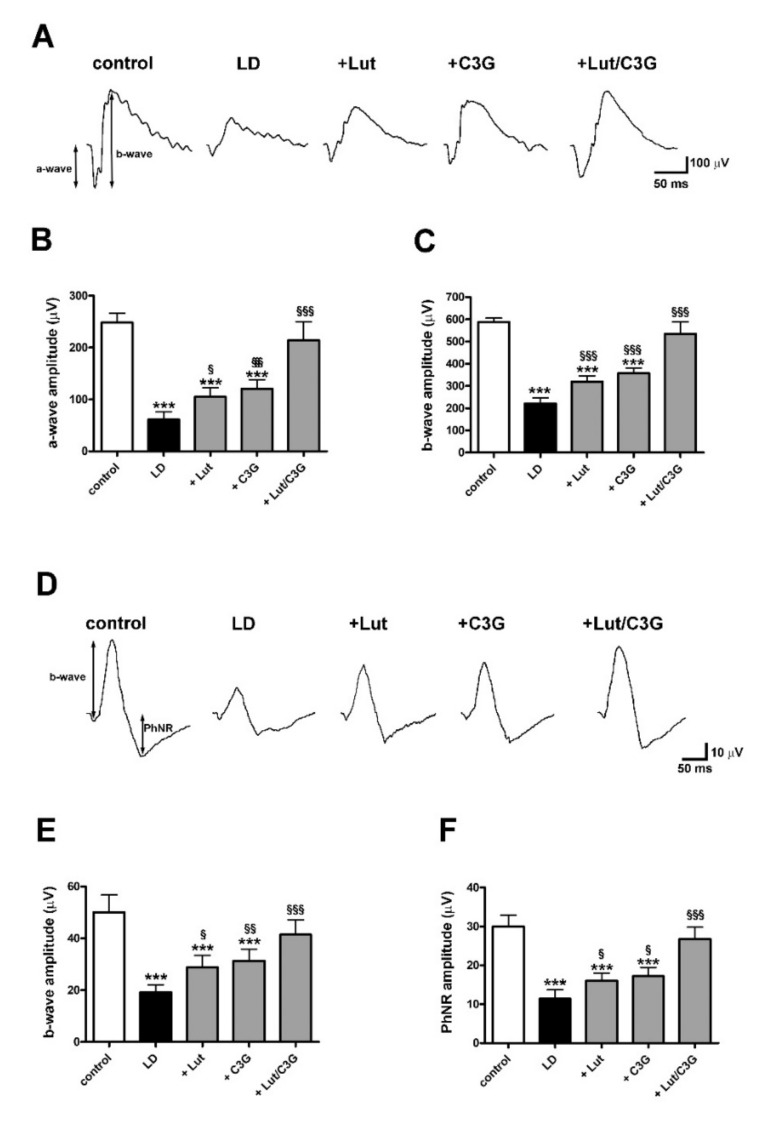
Individual and combined effects of lutein and C3G on scotopic and photopic full field electroretinogram (ERG). (**A**) Representative ERG waveforms showing scotopic a- and b-waves from control and LD rats untreated or pretreated with either lutein, C3G or their combination. (**B**,**C**) Quantitative analysis of scotopic a- and b-wave amplitudes. (**D**) Representative ERG waveforms showing photopic b-waves and photopic negative response (PhNR). (**E**,**F**) Quantitative analysis of photopic b-wave and PhNR amplitudes. Data are expressed as mean ± SD. Differences between groups were tested for statistical significance using one-way ANOVA followed by the Newman–Keuls multiple comparison post hoc test (*n* = 6 animals per group). *** *p* < 0.001 versus control; ^§^ *p* < 0.05; ^§§^ *p* < 0.01; ^§§§^ *p* < 0.001 versus LD.

In additional experiments aimed at evaluating whether protective efficacy of dietary combination on photoreceptor loss were accompanied by ameliorated visual function, LD rats treated with lutein and C3G either alone or in combination were subjected to comprehensive ERG analyses to assess outer and inner retinal function. Under scotopic conditions, the a-wave reflects the activity of rods while the b-wave reflects the activity of bipolar and Müller cells. As shown by scotopic ERG responses in Figure 6A and quantitative analysis of both a- and b-wave amplitudes in Figure 6B,C, in untreated LD rats, displayed a reduced amplitude of a-wave (LD 61.00 ± 15.46, *p* < 0.0001) and b-wave (LD 220.60 ± 25.26, *p* < 0.0001) compared to those measured in controls (control a-wave 248.4 ± 17.95 µV; b-wave 588.20 ± 17.60 µV). The pretreatment with either lutein or C3G administered alone partially preserved the a-wave (+Lut 104.8 ± 17.55 µV, *p* = 0.039; +C3G 120.6 ± 17.42 µV, *p* = 0.0033) and b-wave amplitudes (+Lut 319.4 ± 26.08 µV, *p* = 0.0008; +C3G 357.00 ± 24.36 µV, *p* < 0.0001) compared with untreated LD rats. Lutein in combination with C3G completely preserved the a-wave (+Lut/C3G 214.0 ± 36.01 µV, *p* = 0.14) and b-wave amplitudes (+Lut/C3G 534.40 ± 54.46 µV, *p* = 0.10), resulting in amplitudes similar to those of controls.

Under photopic conditions, additional measurements included the b-wave, which indirectly reflects the cone activity since the a-wave cannot be measured reliably, and the PhNR, a negative-going wave following the b-wave that originates in the inner retinal layer and is correlated with RGC activity [30]. As shown by photopic ERG responses in Figure 6D and quantitative analysis of b-wave and PhNR amplitudes in Figure 6E,F, in untreated LD rats, the photopic b-wave (LD 19.20 ± 2.95 µV, *p* < 0.0001) and the PhNR (LD 11.40 ± 2.30 µV, *p* < 0.0001) were both reduced in amplitude compared to control (control b-wave 50.00 ± 6.78 µV; PhNR 30.00 ± 2.91 µV). Lutein or C3G administered alone partially prevented the photopic ERG dysfunction, with both b-wave (+Lut 28.80 ± 4.60 µV, *p* = 0.047; +C3G 31.40 ± 4.45 µV, *p* = 0.0089) and PhNR amplitudes (+Lut 16.00 ± 2.00 µV, *p* = 0.044; +C3G 17.20 ± 2.28 µV, *p* = 0.013) higher than those in untreated LD rats. The lutein and C3G combination completely preserved the photopic ERG responses in both b-wave (+Lut/C3G 41.40 ± 5.77 µV, *p* = 0.094) and PhNR amplitudes (+Lut/C3G 26.80 ± 3.03 µV, *p* = 0.30) to control levels.

### 3.7. Efficacy of a Pre-Formulated Mixture on Light-Induced Retinal Damage

**Figure 7:**
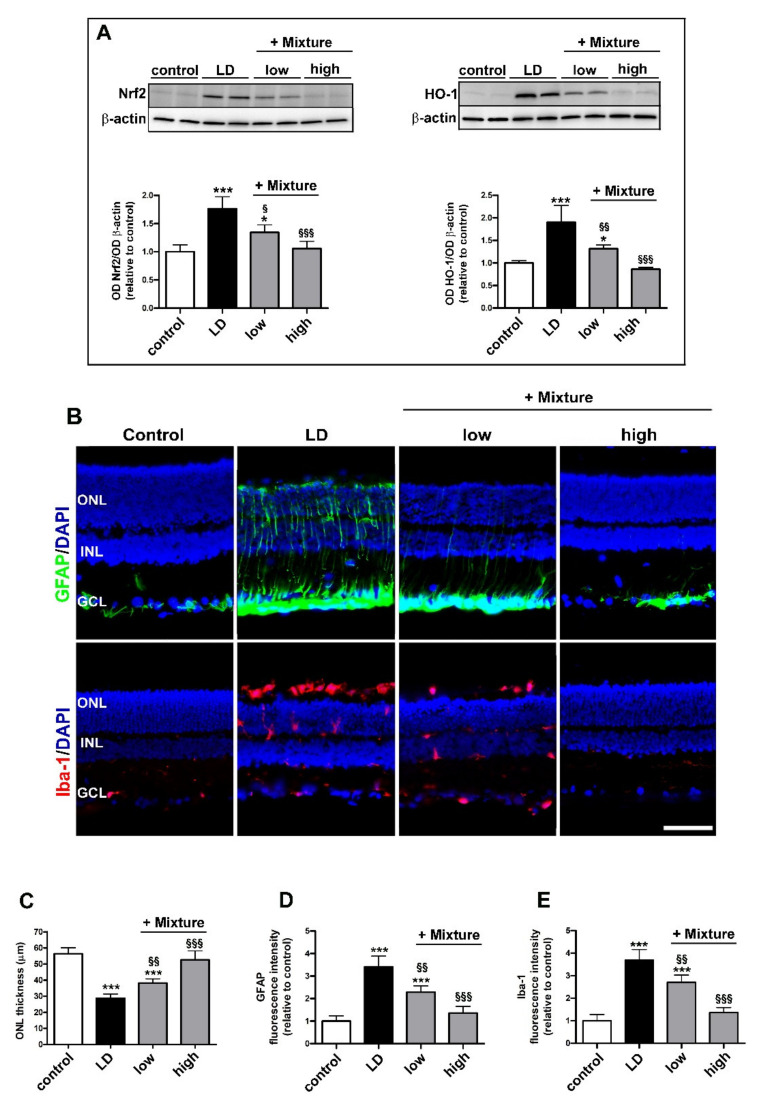
Effects of pre-formulated mixture on oxidative markers, gliosis and microglial activation. (**A**) Western blots and densitometric analysis of Nrf2 and HO-1 in retinal homogenates of control and LD rats untreated or pretreated with the mixture at low or high dosage. (**B**) Representative images of retinal sections immunolabeled for GFAP (green) and Iba-1 (red) and counterstained with DAPI (blue). (**C**) Quantitative analysis of ONL thickness. (**D**,**E**) Quantitative analysis of GFAP and Iba1 immunofluorescence intensity. Scale bar, 50 µm (*n* = 6 retinas per group). Data are expressed as mean ± SD. Differences between groups were tested for statistical significance using one-way ANOVA followed by the Newman–Keuls multiple comparison post hoc test. * *p* < 0.05; *** *p* < 0.001 versus control; ^§^ *p* < 0.05; ^§§^ *p* < 0.01; ^§§§^ *p* < 0.001 versus LD.

After having established the efficacy of the combined treatment of lutein and C3G, we investigated how their effects could be modulated in a more sophisticated blend of functional nutrients. In particular, the efficacy of a mixture including lutein and C3G together with verbascoside and zinc was evaluated at two different doses. As shown in Figure 7A, rats pretreated with a low dose of the mixture showed a significant reduction in upregulated levels of Nrf2 (+Mixture low 1.34 ± 0.13, *p* = 0.010) and HO-1 (+Mixture low 1.31 ± 0.08, *p* = 0.0048) as compared to untreated LD rats. After pretreatment with a high dose of the mixture, the levels of Nrf2 (+Mixture high 1.05 ± 0.13, *p* = 0.95) and HO-1 (+Mixture high 0.86 ± 0.04, *p* = 0.75) did not differ from those measured in controls.

Representative images of retinal sections immunolabeled with GFAP and Iba-1 are shown in Figure 7B, while the quantitative analysis of immunostaining is shown in Figure 7C–E. After low dose of the mixture, the ONL thickness was greater than in untreated LD rats (+Mixture low 38.20 ± 2.58 µm, *p* = 0.0065), while the high dose of the mixture completely prevented the light-induced ONL decrease as compared to control (+Mixture high 52.60 ± 5.59 µm, *p* = 0.42; Figure 7C). As shown in Figure 7D,E, the pretreatment at low dose partially prevented the increase in GFAP (+Mixture low 2.28 ± 0.28, *p* = 0.0023) and Iba-1 immunostaining (+Mixture low 2.71 ± 0.32, *p* = 0.0071) compared to untreated LD rats. After high dose pretreatment, GFAP (+Mixture high 1.35 ± 0.29, *p* = 0.47) and Iba-1 immunostaining (+Mixture high 1.36 ± 0.22, *p* = 0.45) was not different from control rats.

**Figure 8:**
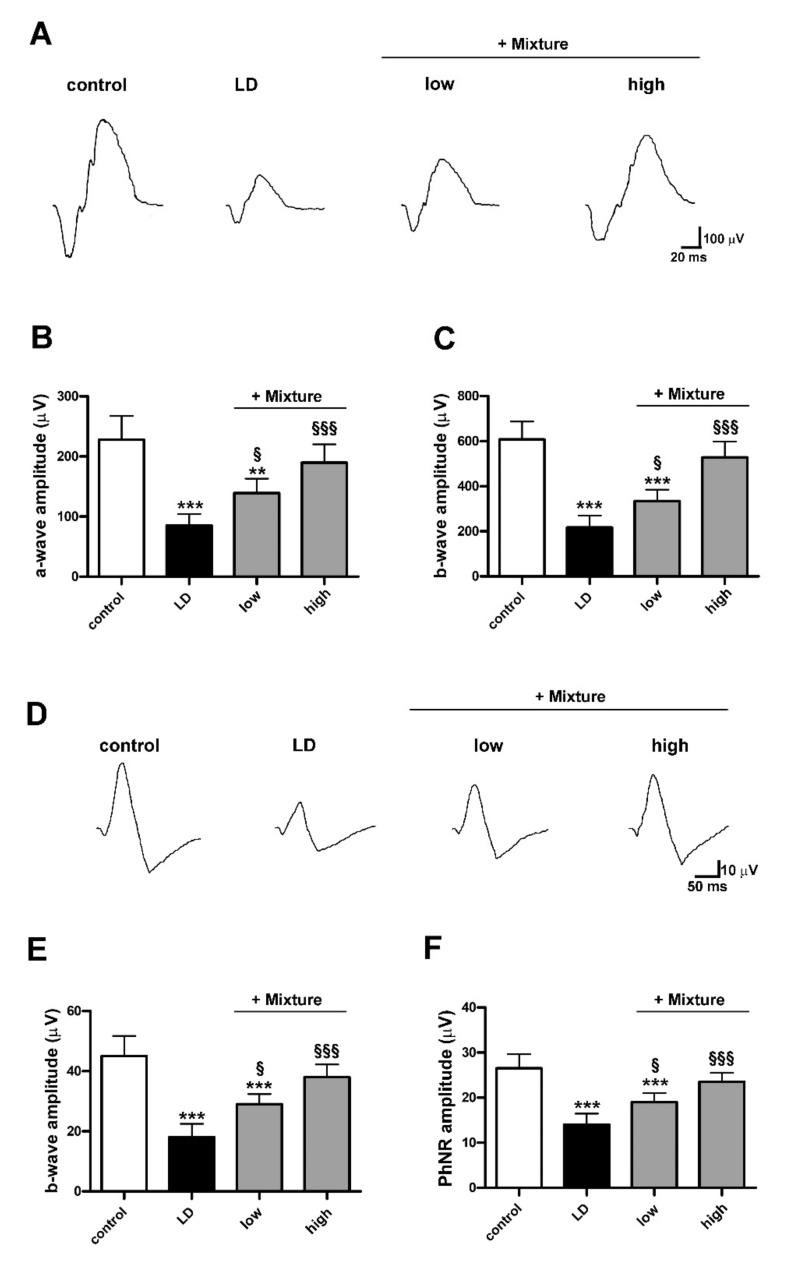
Effects of pre-formulated mixture on scotopic and photopic full field electroretinogram (ERG). (**A**) Representative ERG waveforms showing scotopic a- and b-waves from control and LD rats untreated or pretreated with the mixture at low and high dosage. (**B**,**C**) Quantitative analysis of scotopic a- and b-wave amplitudes. (**D**) Representative ERG waveforms showing photopic b-waves with photopic negative response (PhNR). (**E**,**F**) Quantitative analysis of photopic b-wave and PhNR amplitudes. Data are expressed as mean ± SD. Differences between groups were tested for statistical significance using one-way ANOVA followed by the Newman–Keuls multiple comparison post hoc test (*n* = 6 animals per group). ** *p* < 0.01; *** *p* < 0.001 versus control; ^§^ *p* < 0.05; ^§§§^ *p* < 0.001 versus LD.

Figure 8 shows how light exposure led to a decrease in the amplitude of both scotopic a-wave (LD 85.20 ± 19.23 µV, *p* < 0.0001) and b-wave (LD 216.70 ± 53.22 µV, *p* < 0.0001), and photopic b-wave (LD 18.00 ± 4.47 µV, *p* < 0.0001) and PhNR (LD 14.00 ± 2.46 µV, *p* < 0.0001) as compared to control. The mixture at low dose attenuated the reduction in the amplitude of scotopic (+Mixture low a wave 139.2 ± 23.93 µV, *p* = 0.046; b-wave 334.40 ± 50.54 µV, *p* = 0.048) and photopic waves (+Mixture low b-wave 29.00 ± 3.35 µV, *p* = 0.012; PhNR 19.00 ± 2.01 µV *p* = 0.024) as compared to LD rats. The pretreatment with the mixture at high dose prevented such amplitude reduction maintaining both scotopic (+Mixture high a wave 189.6 ± 30.63 µV, *p* = 0.20; b-wave 527.90 ± 70.66 µV, *p* = 0.025) and photopic ERG responses (+Mixture high b-wave 38.00 ± 4.25 µV, *p* = 0.14; PhNR 23.50 ± 2.01 µV, *p* = 0.25) to control levels suggesting preservation of photoreceptors from death.

## 4. Discussion

Food supplements or dietary interventions including antioxidant compounds are used frequently to prevent retinal diseases [6]. In experimental models of light induced retinal damage, there is indication that diet and dietary supplements with lutein and C3G can be beneficially used against light-associated oxidative stress and inflammatory processes [4,31]. In patients, dietary administration of antioxidants has been shown to reduce the incidence of oxidative stress-related eye diseases [32], although several studies provided conflicting results [33,34]. However, there is little information about whether the combination of lutein and C3G has superior efficacy over a single free supplementation. How their combined efficacy would be modulated in a more sophisticated blend of functional nutrients including additional antioxidant compounds has also been investigated.

### 4.1. Light-Induced Retinal Damage

The eye has evolved several mechanisms to protect the retina from light-induced damage. For instance, macular pigments including lutein confer additional protection to the retina through their ability to absorb high-energy blue light. However, prolonged exposures to high light intensities will cause injury to the eye, a feature that is mimicked in the model of light-induced retinal damage. Exposure to very intense light irradiation for an extended period may result in chemical changes in retinal cells thus ultimately leading to cell death [35]. The importance of light-induced phototoxicity has gained attention in the last years, considering that long-term exposure to video displays and light-emitting diodes may trigger morphological and functional alterations of the retina thus contributing to the increase in the incidence of retinal diseases [36].

The rat model of light damage is characterized by the activation of the Nrf2 antioxidant pathway, as indicated by the present results and previous findings [37,38]. This is a homeostatic response aimed at counteracting the light-induced overproduction of ROS in the retina. However, in pathological conditions, ROS production overcomes the endogenous antioxidant capacity, thus promoting the onset of oxidative stress that, in turn, triggers inflammation, gliosis and microglia activation culminating in the apoptotic death of retinal cells [39]. The activation of the apoptotic cascade is paralleled by photoreceptor loss as determined by a drastic decrease in ONL thickness. As a consequence of photoreceptor loss, both scotopic and photopic ERG responses are reduced in amplitude thus indicating a drastic impairment of the visual function, in line with previous reports [26].

### 4.2. Preventive Efficacy of Antioxidant Compounds

With a global increasing rate of light-induced degenerative diseases of the retina, it is important to develop preventive methods to mitigate light-related manifestations and curb the progression of retinal damage. In this respect, dietary regimens rich in antioxidant compounds including lutein or C3G have been reported to actively counteract light-induced retinal damages. In particular, lutein plays a crucial role acting not only as an important regulator of redox balance, but also as a structural molecule in cell membranes and short wavelength light filter in retinal tissues [40]. Similarly, C3G has been established to act as a stabilizer of free radicals by its hydrogen donating ability and also to promote the regeneration and synthesis of rhodopsin [41]. As shown by the present findings, after dietary administration, lutein and C3G are detected in the plasma and ocular tissues in line with previous results [42,43]. The evaluation of lutein and C3G in ocular tissues has been preliminary to the analysis of their efficacy since it allowed to determine whether each compound effectively reaches the ocular tissues where their efficacy to prevent light-induced damage has been tested. Several studies suggest that high blood levels of lutein or C3G are associated with reduced risk of ocular diseases suggesting the beneficial effects of their dietary intake [44,45]. Since lutein and C3G cannot be synthesized by the human body, their plasma levels can only be ensured through food intake or dietary supplements. They are delivered to the retina by the choroidal vascular plexus. In the retina, we found lutein levels higher than those of C3G although their choroidal levels were similar. Reduced levels of C3G in the retina can be attributed to its degradation leading to the formation of the corresponding phenolic acids [46]. Lutein and C3G may exert their antioxidant activity acting as both radical scavengers and antioxidant defense inducers via Nrf2 activation [47,48]. However, how antioxidants preferentially act as radical scavengers or Nrf2 inducers is yet to be elucidated. In our experimental conditions, the LD-triggered increase in Nrf2 and HO-1 levels, demonstrating the activation of the antioxidant system, was almost prevented by the pre-treatment with Lutein and C3G. This is likely to derive from the accumulation of both compounds in the retina following their 7-days preventive administration which allowed to buffer the LD-driven increase in free radical formation and decreasing the upstream ROS-driven activation of the Nrf2-HO-1 pathway. However, the contribution of a preventive activation of the Nrf2 antioxidant pathway further preventing the LD-driven ROS accumulation cannot be excluded.

Reduced activity of Nrf2/HO-1 correlates with the inhibition of inflammatory processes, as demonstrated by the decreased phosphorylation of NF-kB and the partial recovery of cytokine levels. The antioxidant and the anti-inflammatory properties of lutein or C3G result in effective but incomplete preservation of both rods and cones that is reflected in ameliorated visual function, in line with previous findings [4,49]. Lutein or C3G, when administered alone, similarly ameliorate the pathological signs of retinal damage. In contrast, their combined administration provides synergistic efficacy to protect the retina from light damage. In particular, lutein and C3G exert a multitarget role by hampering major retinal events activated by light, as demonstrated by the general reduction in upregulated levels of oxidative and pro-inflammatory markers associated with neuroprotective effects and recovered visual function. In particular, the finding that the combined administration of lutein and C3G preserves scotopic and photopic ERG is indicative of its neuroprotective efficacy on both photoreceptor and post-receptor cells, including bipolar cells and retinal ganglion cells [50].

Multicomponent formulae of antioxidant compounds, which are available without a prescription, are widely used by many people as preventive therapy for eye diseases. Several studies support the protective role of nutraceutical mixtures in ocular health although their benefits as putative therapeutical strategies are still limited and sometimes controversial. Different mixtures of antioxidant nutrients have been proven to protect the retina from light, although superior efficacy of a combination over a single free supplementation is not obvious [51]. In fact, the total antioxidant capacity of a complex mixture is likely to depend on either the antagonistic or synergistic interaction among the different compounds. In particular, the antagonism occurs when the sum of the effects is less than the mathematical sum that would be predicted from individual components [52]. On the other hand, the combination of antioxidant compounds with complementary action may play an important role to optimize their action. For instance, in the rd10 mouse model of retinitis pigmentosa, combined antioxidant compounds positively interact with each other to ameliorate retinal functionality and morphology [53]. In this respect, our focus on photoreceptors as the main targets of light damage and Müller cells as targets of inflammation with a prominent expression of GFAP and Iba-1 by microglial cells, does not exclude the involvement of additional cell types. In particular, both Müller cells and ON-center bipolar cells are involved in the generation of the b-wave (ON-center bipolar hypothesis) and they are affected by light exposure as a consequence of photoreceptor damage [54]. Here, we provide the first evidence that a mix of antioxidant compounds including zinc and verbascoside in addition to lutein and C3G, effectively protects photoreceptor cells against light-induced damage thus preventing some secondary changes that are likely to be induced in the inner retina, as demonstrated by restored ERG. In this respect, the extracellular currents that generate the b-wave either originate directly in ON-bipolar cells or reflect potassium-induced changes in the membrane potential of Müller cells enveloping them. In addition, the b-wave is also affected by light-induced activity in third-order retinal neurons (amacrine cells and RGCs) as demonstrated by the PhNR, a negative-going wave following the b-wave that originates in the inner retinal layer in response to photopic conditions and is correlated with RGC activity [30].

In addition to the protective efficacy of lutein in combination with C3G, zinc and verbascoside have been reported individually as active compounds against retinal diseases. Zinc is an essential microelement that plays a main role in maintaining normal ocular function by serving as an antioxidant [55], and its supplementation is reported to slow down the progression of age-related macular degeneration [56]. Verbascoside is a glycoside present in many medicinal herbs and possesses anti-oxidative, anti-inflammatory and neuroprotective properties [57]. Despite its poor bioavailability, there is evidence that diet supplementation with verbascoside reduces oxidative stress in ocular tissues and fluids, while in vitro verbascoside inhibits apoptotic death of retinal cells [58,59]. The synergistic effects of antioxidant mixtures may increase the bioavailability of partnering compounds, further improving treatment outcomes. In addition, the bioactivity of each individual component may occur at different levels of the same signaling cascade or activate different pathways thus leading to multi-target and multifunctional effects [21].

## 5. Conclusions

**Figure 9:**
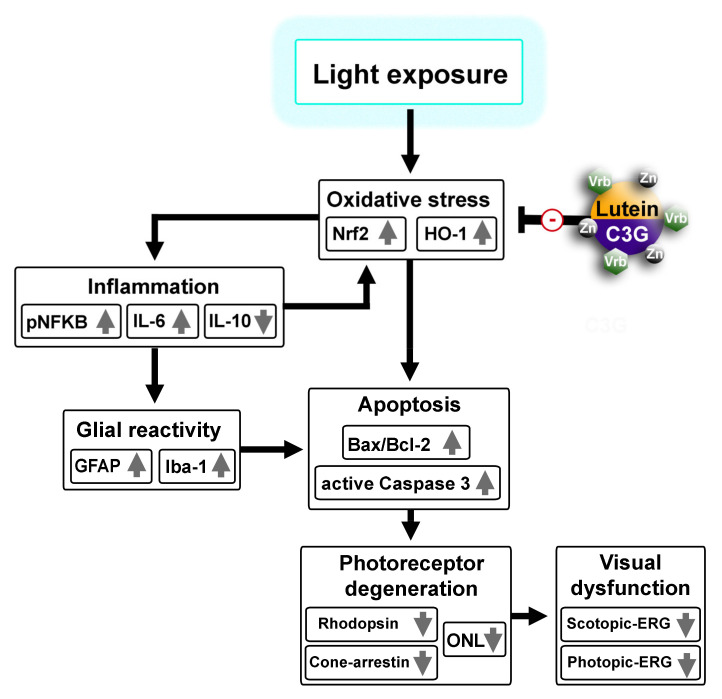
Schematic diagram showing possible mechanisms through which a nutraceutical blend containing lutein, cyanidin-3-glucosid (C3G), zinc (Zn) and verbascoside (Vrb) prevents light induced oxidative stress and the downstream cascade leading to photoreceptor degeneration and visual dysfunction.

Taken together, the present evidence that combined administration of lutein, C3G, verbascoside and zinc successfully ameliorates retinal dysfunction by acting as a major antioxidant compound can now offer new perspectives not only for implementing our knowledge on the effectiveness of dietary supplements in counteracting light-induced retinal damage, but also for eventually expanding complementary nutritional interventions. As shown in the schematic representation of Figure 9, the present data demonstrate the ability of lutein, C3G, verbascoside and zinc to preserve visual function by preventing retinal cell apoptosis through a major antioxidant action leading to reduced levels of pro-inflammatory markers. The beneficial efficacy of the multicomponent formula would also encourage the development and consumption of foods and/or supplements rich in these compounds.

## Figures and Tables

**Table 1:** Primary antibodies list used in the Western blot analysis.

Antibody	Dilution	Source	Catalogue
Rabbit monoclonal anti-Bax	1:500	Abcam	ab182733
Rabbit polyclonal anti-Bcl-2	1:500	Abcam	ab194583
Rabbit polyclonal anti-cleaved caspase 3	1:500	Abcam	ab2302
Rabbit monoclonal anti-Nrf2	1:300	Abcam	ab62352
Rabbit polyclonal anti-HO-1	1:500	Abcam	ab13243
Rabbit polyclonal anti-pNF-kB p65 (Ser 536)	1:100	Santa Cruz Biotechnology	sc-33020
Rabbit polyclonal anti-NF-kB p65	1:1000	Abcam	ab16502
Mouse monoclonal anti-IL-6	1:100	Santa Cruz Biotechnology	sc-57315
Goat polyclonal anti-IL-10	1:100	Santa Cruz Biotechnology	sc-1783
Mouse monoclonal anti-β-actin	1:2500	Sigma-Aldrich	A2228

## Data Availability

The data presented in this study are available on request from the corresponding author.
